# Differences in prostate cancer treatment receipt and timeliness of treatment between African American/Black and White Iowans

**DOI:** 10.1007/s10552-025-02064-6

**Published:** 2025-10-13

**Authors:** Gawain J. Williams, Whitney E. Zahnd

**Affiliations:** 1 Department of Health Management and Policy, College of Public Health, University of Iowa, 145 N. Riverside N271, Iowa City, IAIowa 52242 USA; 2 Holden Comprehensive Cancer Center, University of Iowa, Iowa City, Iowa USA

**Keywords:** Cancer registry, Treatment disparities, Prostate cancer disparities, Racial disparities

## Abstract

**Background:**

Non-Hispanic Black (NHB) men face higher prostate cancer (PCa) mortality rates compared to other racial/ethnic groups. Factors contributing to these disparities, particularly concerning healthcare system factors, remain uncertain. We investigate differences in treatment receipt and timeliness between NHB, non-Hispanic White (NHW) and Hispanic Iowans, examining variations across treatment facilities.

**Methods:**

Demographic, tumor, treatment, and hospital characteristics of PCa patients 40–99 years were obtained from the Iowa Cancer Registry (2010–2020). We used logistic regression to estimate the odds of receiving definitive treatment, time to treatment, and the type of treatment received.

**Results:**

Among 18,747 PCa patients, 18,197 (97.1%) were NHW, 550 (2.9%) NHB, and 155 Hispanic (0.83%). NHB men were younger. The odds of receiving definitive treatment among advanced stage patients were significantly lower for NHBs compared to NHWs (adjusted odds ratio-AOR- 0.39; 95% CI 0.26—0.59). More NHBs underwent PCa treatment at either NCI-designated or CoC-accredited facilities. NHBs receiving care at an NCI-designated center or at a non-accredited center were less likely to receive definitive treatment compared to NHWs. Furthermore, NHBs with advanced PCa had diminished odds of receiving definitive treatment regardless of the status of the treatment facility (NCI: AOR = 0.34; 95% CI 0.13 – 0.93; CoC only: AOR = 0.47; 95% CI 0.29 – 0.76; Neither: AOR 0.24; 95% CI 0.10–0.60).

**Conclusion:**

NHB men with an advanced staging had lower odds of receiving definitive treatment across treatment settings. Further research and intervention are needed to reduce these disparities and improve PCa outcomes.

**Supplementary Information:**

The online version contains supplementary material available at 10.1007/s10552-025-02064-6.

## Introduction

Prostate cancer (PCa) accounts for nearly 15% of all new cancer diagnoses within the United States, with an estimated 299,010 new cases expected in the year 2024 [1]. Additionally, it ranks as the second leading cause of cancer-related mortality [2]. While the five-year survival rate of PCa is high, it is not consistent across all populations [3]. Between the years 2000 and 2015, Non-Hispanic White (NHW) men had a relative survival of 98.2%, Non-Hispanic Black (NHB) 95.99%, American Indian/Alaska Native 94.67%, and Asian or Pacific Islanders had a relative survival of 95.63% [4]. NHB men have a greater incidence rate and two times the likelihood of PCa-related mortality than that of NHW men [5–9]. Furthermore, NHB men are more likely to be diagnosed at a younger age and with a more advanced and aggressive disease, leading to higher rates of mortality [10–12]. Additionally, NHB men exhibit higher prostate-specific antigen (PSA) levels and higher Gleason scores compared with NHW men [10, 13].

A growing body of evidence points to differences in the receipt and timeliness of treatment received by members of various racial/ethnic groups that may contribute to mortality and survival disparities [14]. NHB PCa patients often receive fewer treatment options, are less likely to undergo definitive therapy (radical prostatectomy, local destruction, beam radiation and or brachytherapy), and are more likely to receive no treatment at all when compared to their white counterparts [10, 15–17]. This is particularly noteworthy considering NHB individuals often experience a more aggressive disease [10]. This subsequently leads to NHB PCa patients having a higher PCa mortality rate than NHW patients [6, 10, 18].

The type of facility where an individual receives care may also play a role in receipt of treatment and, ultimately, long-term outcomes [19–22]. The National Cancer Institute’s (NCI) cancer center designation program designates cancer centers nationwide which, within stringent criteria, provide transdisciplinary, cutting-edge cancer research aimed at innovating strategies for cancer prevention, diagnosis, and treatment [23]. These designated centers offer access to innovative treatments and care options which may not be available elsewhere. Prior research found that patients treated at NCI-designated centers had lower adjusted surgical mortality rates and superior survival when compared to non-designated hospitals [19, 24–26]. Similarly, the American College of Surgeons’ Commission on Cancer (CoC) accreditation has also been essential in the advancement of high-quality cancer treatment. The CoC, a consortium of professional organizations, is dedicated to improving cancer care survival and quality. To earn CoC accreditation, a cancer care facility must meet rigorous quality-of-care standards, as well as be reassessed triennially to ensure that a high standard is maintained [27]. Health care facilities which boast either of these designations or accreditations are considered to be high-quality treatment facilities within their cancer care field.

Iowa ranks among the top five states nationally for the highest incidence rate of PCa cases per 100,000 people.[28]. In Iowa, the most notable racial disparities in age-adjusted mortality are observed in PCa-related deaths [11]. In Iowa, NHB men face a mortality risk from PCa approximately 1.8 times higher than that of NHW men. This stark inequity highlights the critical need for additional research to understand the factors contributing to these disparities in Iowa. It remains unclear whether factors within the healthcare system, such as the timing and quality of treatment, contribute to the unequal outcomes experienced by NHB men diagnosed with PCa in Iowa. Therefore, our objective is to identify if there are variations in the treatment received and the timeliness of treatment between NHB and NHW patients within the state.

## Materials and methods

### Data source

Data were obtained from the Iowa Cancer Registry (ICR). The ICR is a population-based cancer registry affiliated with the National Cancer Institute’s Surveillance, Epidemiology, and End Results (SEER) Program, which has gathered information on all cancer diagnoses of Iowa residents since 1973. Data were extracted for all men between the ages of 40 years and 99 years inclusive of any race, with a malignant PCa diagnosed between the years 2010 and 2020 [29, 30]. Our study does not include those who were diagnosed at autopsy or on death certificate only, were a lab-only case, or received treatment only within the Veteran Affairs system. We further excluded individuals who were not linked to a hospital or treatment facility. This study was approved by the Institutional Review Board at the University of Iowa.

### Independent variables and covariates

Our main independent variable of interest was a patient’s race/ethnicity which we categorized as either NHB, NHW, or Hispanic. Independent variables related to facility, including distance to treatment facility, type of treatment facility, and rural–urban designation of treating facility, were also considered. Distance to treatment facility is important as that distance to treatment facility may influence the type of treatment received [31–33]. Characteristics of the treatment facility, such as NCI [23] designation, and CoC[27] accreditation, were also included as these factors may influence the time to care and the quality of care. We considered the rurality of the cancer treatment facility by using the Rural–Urban Continuum Code (RUCC) of the county in which the hospital is located to distinguish if the facility is in a metropolitan or nonmetropolitan area. RUCCs distinguish U.S. metropolitan counties by the population size of their metro area and nonmetropolitan counties by their degree of urbanization and adjacency to a metropolitan area [34]. Treatment facilities in nonmetropolitan areas often have a limited number of general practitioners and specialized professionals, such as oncologists, radiologists, pathologists, surgeons, and radiation oncologists [31, 35, 36].

We also considered patient characteristics including age, insurance status, marital status, rural/urban residence, and year of diagnosis. Patients with unknown cancer stage were excluded from all regression analyses but retained in descriptive tables. Marital status was included in the regression models as a categorical variable “with unknown treated as its own category. Marital status categorization encompassed married (inclusive of legal, common law, and unmarried domestic partner), separated/divorced/widowed, and single, never married, and unknown. Insurance statuses included Medicaid (Medicare, private insurance, and governmental/military plans (encompassing TRICARE, military sponsored plans, Veterans Affairs, and Indian Health Service plans).

Clinical characteristics included cancer staging (I, II, III, IV) and the numbers of days from diagnosis to the start of each treatment option. PCa staging was based on the Derived AJCC Stage Group, 7th edition (2010–2017 patients)[37] and Derived EOD 2018 Stage Group (2018 + patients)[38] dependent upon when the patient was diagnosed. Given the differences in treatment recommendations[39] based on the staging of the cancer, we stratified cancer staging into two groups: early stage and advanced stage. Cancer stages I and II were categorized as early stage, and stages III and IV were categorized as the advanced stage. Gleason scores were categorized into three clinically meaningful risk groups, namely: low grade (Gleason scores of 6 or lower), intermediate grade (Gleason score of 7), and high grade (Gleason score of 8 or higher) in accordance with the American Cancer Society’s categorization [40]. While the five-tier International Society of Urological Pathology (ISUP) grade group system offers more granular data and has seen increased adoption in use in recent years, this level of detail was not available across the entire study period. Additionally, since the Gleason score is used as a covariate to control for disease severity and not a primary exposure within the study, the three-tier grouping is sufficient for our model and for interpretability and to ensure model stability across years.

### Outcomes

We examined two key outcomes: the likelihood of receiving definitive treatment, as well as the likelihood of receiving definitive treatment within 90 days after diagnosis. Definitive treatment was defined as the receipt of either radical prostatectomy or radiation therapy, including local destruction or brachytherapy, or any combination of these modalities. These interventions were identified due to their intent to treat PCa with curative purpose [41, 42]. Furthermore, within our analysis, we stratified the data based on the type of definitive treatment (surgery vs. radiation) to explore potential statistical differences in the timing of each treatment modality. A commonly employed timeframe for evaluating the timeliness of treatment initiation disparities is within ninety days following the initial diagnosis [43, 44]. A prior study has suggested that delays in radiation treatment for high-risk prostate cancer (PCa) patients result in less favorable health and quality of life outcomes [45]. Additionally, research further indicates a significant increase in the risk of biochemical recurrence among patients who experienced a delay post 90 days after diagnosis to start of curative treatment[46] In that study, these outcomes were identified in intermediate-risk patients with delayed prostatectomy, with no differences observed among low-risk patients [46]. We also sought to find if there were differences in the time to receipt of adjuvant treatment among those who received definitive treatment. We focus only on those who received adjuvant radiotherapy after prostatectomy, as it is considered the standard of care for patients with high-risk pathology or rising PSA values [47].

### Statistical analysis

Descriptive statistics were calculated for the study cohort, encompassing means and standard deviations for continuous variables, and frequencies and percentages for categorical variables. Logistic regression analysis was conducted to assess the odds of receiving specific treatment modalities, accounting for patient demographics, clinical attributes, and healthcare facility factors. Additionally, logistic regression was employed to evaluate the odds of undergoing any type of definitive therapy within the initial 90-day period following diagnosis. Odds ratios and absolute risk differences are reported. We defined two-sided statistical significance as *p* < 0.05. All statistical analyses were completed using STATA 17.0. The study was approved as an exempt study under the University of Iowa institutional review board.

## Results

Of the 18,747 in our sample, 550 (2.93%) were NHB, 18,197 (97.07%) NHW, and 155 (0.83%) identified as being of Hispanic ethnicity (Table 1). A greater proportion of NHB men, as well as men of Hispanic ethnicity, were diagnosed at an earlier age than NHW men. On average, most NHB patients received a diagnosis between 50 and 64 years old, while most NHW men received a diagnosis at age 65 years and older.

**Table 1:** Descriptive Summary of Full Patient Population, Iowa Cancer Registry

	Total (*n* = 18,747), No.(%)	NH Black (*n* = 550 [2.93%]), No. (%)	NH White (*n* = 18,197 [97.07%]), No.(%)	Hispanic (*n* = 155 [0.83%]), No.(%)
**Age at Diagnosis**				
< 50	273 (1.46)	26 (4.73)	247 (1.36)	< 10 (< 6.45)
50—64	7,295 (38.96)	306 (55.64)	6,989 (38.41)	71 (45.81)
65—74	7,589 (40.48)	177 (32.18)	7,412 (40.73)	58 (37.42)
75 +	3,590 (19.15)	41 (7.45)	3,549 (19.50)	21 (13.55)
**Marital Status**				
Married	14,150 (75.48)	287 (52.18)	13,863 (76.18)	115 (74.19)
Separated	2,294 (12.24)	96 (17.45)	2,198 (12.08)	18 (11.61)
Single	1,640 (8.75)	148 (26.91)	1,492 (8.20)	18 (11.61)
Unknown	663 (3.54)	19 (3.45)	644 (3.54)	< 10 (< 6.45)
**Insurance Status**				
Medicaid	358 (1.91)	65 (11.82)	293 (1.61)	11 (7.10)
Medicare	9,858 (52.58)	209 (38.00)	9,649 (53.03)	62 (40.00)
Government/ Military	774 (4.13)	43 (7.82)	731 (4.02)	< 10 (< 6.45)
Insurance, NOS	1,397 (7.45)	53 (9.64)	1,344 (7.39)	14 (9.03)
Private	5,741 (30.62)	156 (28.36)	5,585 (30.69)	54 (34.84)
None/Unknown	619(3.30)	24 (4.36)	595 (3.27)	< 10 (< 6.45)
**Patient Rurality**				
Metropolitan	9,513 (50.74)	475 (86.36)	9,038 (49.67)	94 (60.65)
Non-Metropolitan	9,234 (49.26)	75 (13.64)	9,159(50.33)	61 (39.35)
**Cancer Stage**				
I	2,697 (14.39)	58 (10.56)	2,639 (14.50)	19 (12.26)
II	9,954 (53.10)	296 (53.92)	9,658 (53.07)	61 (39.35)
III	3,030 (16.16)	88 (16.03)	2,942 (16.17)	38 (24.52)
IV	2,212 (11.80)	87 (15.85)	2,125 (11.68)	28 (18.06)
Unknown	853 (4.55)	20 (3.64)	833 (4.58)	< 10 (< 6.45)
**Gleason Score**				
Low risk	4,596 (24.52)	104 (18.91)	4,492 (24.69)	31 (20.00)
Intermediate Risk	8,112 (43.27)	266 (48.36)	7,846 (43.12)	66 (42.58)
High Risk	4,698 (25.06)	144 (26.18)	4,554 (25.03)	43 (27.74)
No Needle core biopsy/Turp performed	1,097 (5.85)	34 (6.18)	1,063 (5.84)	12 (7.74)
Unknown	244 (1.30)	< 10 (< 6.45)	242 (1.33)	< 10 (< 6.45)
**Treatment Facility Attended**				
NCI	3,107 (16.57)	117 (21.27)	2,990 (16.43)	28 (18.06)
CoC	9,430 (50.30)	303 (55.09)	9,127 (50.16)	79 (50.97)
Non CoC	6,210 (33.13)	130 (23.64)	6,080 (33.41)	48 (30.97)
**Rurality of Treatment Facility Attended**				
Metropolitan	15,245 (81.32)	516 (93.82)	14,729 (80.94)	140 (90.32)
Non-Metropolitan	3,502 (18.68)	34 (6.18)	3,468 (19.06)	15 (9.68)
**Year of Diagnosis**				
2010	1,678 (8.95)	25 (4.55)	1,653 (9.08)	< 10 (< 6.45)
2011	1,780 (9.49)	35 (6.36)	1,745 (9.59)	12 (7.74)
2012	1,502 (8.01)	25 (4.55)	1,477 (8.12)	< 10 (< 6.45)
2013	1,405 (7.49)	45 (8.18)	1360 (7.47)	< 10 (< 6.45)
2014	1,427 (7.61)	45 (8.18)	1,382 (7.59)	19 (12.26)
2015	1,532 (8.17)	48 (8.73)	1,484 (8.16)	18 (11.61)
2016	1,670 (8.91)	49 (8.91)	1,621 (8.91)	13 (8.39)
2017	1,851 (9.87)	57 (10.36)	1,794 (9.86)	15 (9.68)
2018	1,873 (9.99)	59 (10.73)	1,814 (9.97)	13 (8.39)
2019	2,121 (11.31)	76 (13.82)	2,045 (11.24)	17 (10.97)
2020	1,908 (10.18)	86 (15.64)	1,822 (10.01)	22 (14.19)

NCI, National Cancer Institute Designated; COC, Commission on Cancer Accreditation

Across all three groups, Medicare and private insurances were the most common insurance plans for PCa patients (Table 1). Most PCa patients were from metropolitan areas, in which the majority of NHB and Hispanic patients resided. NHW, on the other hand, showed more balance in geographical dispersion, as 49.67% lived in metro areas. Most NHB and NHW were diagnosed at an early stage, as 64% (354 patients) of all NHB and 68% (12,297) NHW patients had a cancer diagnosis to be within stage one or two. Those with Hispanic ethnicity, however, had the largest proportion (18.06%) of patients with a stage IV diagnosis. Among NHB patients, 87 (16%) of patients had a stage IV diagnosis. Among the NHW population, 2,125 or 12% of the population had a diagnosis of stage IV.

### Definitive treatment

The distribution of patients varied by treatment facility type. Specifically, more NHB patients (21%) received care at an NCI and CoC-accredited facility than NHW patients (17%), or Hispanic patients (14%) (Table 2). More than half (55%) of patients received definitive treatment at a CoC-accredited facility. A larger proportion (63%) of NHB men received definitive care at CoC-accredited facilities compared to 55% of NHW and 51% of Hispanic patients. Contrastingly, 35% of Hispanic men, 29% of NHW men, and 16% of NHB men received definitive care for PCa at facilities which had neither NCI designation nor CoC accreditation.

**Table 2:** Summary of patients who received definitive treatment

	Total (*n* = 12,223), No.(%)	NH Black (*n* = 337 [2.76%]), No. (%)	NH White (*n* = 11,886 [97.24%]), No.(%)	Hispanic (n = 98 [0.80%]), No.(%)
**Treatment Facility**				
NCI	2,050 (16.77)	73 (21.27)	1,977 (16.63)	14 (14.29)
COC	6,755 (55.26)	212 (62.91)	6,543 (55.05)	50 (51.02)
Non-COC	3,418 (27.96)	52 (15.43)	3,366 (28.32)	34 (34.69)
**Rurality of Patients**				
Metropolitan	6,595 (53.96)	298 (88.43)	6,297 (52.98)	59 (60.20)
Non-Metropolitan	5,628 (46.04)	39 (11.57)	5,589 (47.02)	39 (39.80)
**Rurality of Cancer Care center**				
Metropolitan	10,594 (86.67)	326 (96.74)	11,886 (86.39)	90 (91.84)
Non-Metropolitan	1,629 (13.33)	11 (3.26)	1,618 (13.61)	< 10 (< 10.00)
**Average Distance Traveled to Cancer Care Facility to received Definitive Care**				
NCI	88.95 miles (SD 59.95)	71.30 miles (SD 51.34)	89.60 miles (SD 60.15)	52.86 miles (SD 34.21)
COC	23.50 miles (SD 23.94)	7.79 miles (SD 17.47)	24.01 miles (SD 23.95)	17.37 miles (SD 24.90)
Non-COC	22.51 miles (SD 20.74)	17.83 miles (SD 25.81)	22.58 miles (SD 28.65)	12.63 miles (SD 16.26)
Average Distance Traveled to Any Cancer Center to Receive Definitive Care	34.20 miles (SD 40.54)	23.09 miles (SD 38.91)	34.51 miles (SD 40.55)	20.80 miles (SD 27.16)

NCI, National Cancer Institute Designated; COC, Commission on Cancer Accreditation

A greater proportion of patients who received definitive care resided in metropolitan areas within the state of Iowa. Of the total 12,223 patients who received definitive care, 53.96% lived in a metropolitan area. 298 (88.43%) of NHB patients who received definitive treatment resided in a metropolitan area. A notable shift was observed within the NHW patient subgroup. While most patients in the overall cohort resided in nonmetropolitan areas, a larger proportion of NHW patients who received definitive treatment were from metropolitan areas (Table 2).

There was a very metropolitan-centric attendance for patients seeking definitive treatment. 86.67% of the definitive treatment subgroup received treatment at a metropolitan-located treatment facility. NHB patients (96.74%) were the largest proportion of patients to receive definitive treatment at a facility located within a metropolitan area. 86.39% of NHW patients received care at a metropolitan-based facility. 91.84% of the Hispanic population who received definitive care received care at a facility based in a metropolitan area.

In general, patients seeking definitive care traveled the greatest distances to treatment facilities that possessed both NCI designation and CoC accreditation. The overall average distance traveled to an NCI & CoC facility was 88.95 miles (SD 59.95) (Table 2). Specifically, NHW patients traveled further to receive care at a facility with both accreditation and designation at 89.60 miles (SD 60.15). NHB patients traveled on average 71.30 miles (SD 51.34) to receive definitive care at an organization which has both NCI designation and CoC accreditation. Hispanic patients on average traveled approximately 52.86 miles (SD 34.21) to receive care at a facility with both statuses. The average distance traveled to receive definitive care at a facility with only CoC accreditation was 23.5 miles (SD 23.94). On average, all race/ethnic groups traveled shorter distances to receive definitive care at a CoC facility compared to a facility which boasts both NCI & CoC status. NHB patients on average traveled 7.79 miles (SD 17.47), while NHW on average traveled 24.01 miles (23.95), with Hispanic patients traveling on average 17.37 miles (SD 24.90). On average, those who received definitive treatment at facilities with neither NCI nor CoC status traveled 22.51 miles (SD 20.74) with NHB patients traveling 17.83 miles (SD 25.81) NHW 22.58 miles (SD 28.65), and Hispanic patients traveling on average 12.63 miles (SD 16.26).

### Likelihood of receiving definitive treatment

Within the early stage subgroup, there were no statistically significant differences in the likelihood of NHB patients receiving treatment when compared to NHW early stage patients (Table 3). Within the advanced stage subgroup, NHB patients were less likely to receive definitive treatment among NHW advanced stage patients (adjusted OR 0.39; 95%CI, 0.26 – 0.59).

**Table 3:** Multivariable analysis of predictors of receiving definitive treatment among NHB men when compared to NHW

	NHB compared to NHW		Hispanic compared to Non-Hispanic White	
	Early Stage (n = 12,342)	Advanced Stage (n = 5,187)	Early Stage (n = 12,342)	Advanced Stage (n = 5,187)
	OR (95%CI)			
**Race/Ethnicity**				
NHW	*ref*			
NHB	0.70 (0.49 – 1.01)	0.39* (0.26—0.59)		
Non-Hispanic			*ref*	
Hispanic			1.05 (0.54 – 2.03)	0.36* (0.18 – 0.70)
**Age Groups**				
< 50	*ref*			
50–64	0.77 (0.52—1.15)	0.85 (0.41—1.79)	0.66 (0.42—1.04)	0.85 (0.41—1.77)
65–74	0.41* (0.27—0.62)	0.53 (0.25—1.14)	0.37* (0.23—0.58)	0.55 (0.26—1.18)
75 +	0.14* (0.94 – 0.22)	0.10* (0.04—0.21)	0.08* (0.05—0.13)	0.10* (0.05—0.22)
**Marital Status**				
Married	*ref*			
Separated/Divorced/Widowed	0.67* (0.57—0.78)	0.72* (0.57—0.89)	0.67* (0.57—0.78)	0.70* (0.57—0.88)
Single, Never Married	0.63* (0.53—0.75)	0.54* (0.41—0.69)	0.62* (0.52—0.74)	0.51* (0.40—0.66)
Unknown	0.75* (0.56—0.99)	1.04 (0.63—1.71)	0.75* (0.56—1.00)	0.98 (0.60—1.60)
**Insurance Status**				
Government Military	*ref*			
Insurance, NOS	2.41* (1.74—3.34)	2.01* (1.14—3.56)	2.43* (1.75—3.36)	2.06* (1.17—3.63)
Medicaid	1.82* (1.18—2.82)	0.75 (0.42—1.34)	1.78* (1.14—2.76)	0.70 (0.39 -1.26)
Medicare	1.68* (1.32—2.15)	1.20 (0.82—1.77)	1.69* (1.33—2.17)	1.21 (0.82—1.78)
Private	2.33 * (1.80—3.03)	1.50 (0.98—2.30)	2.35* (1.82—3.05)	1.56* (1.02—2.38)
None/Unknown	1.95* (1.35—2.83)	0.70 (0.39—1.24)	1.96* (1.36—2.84)	0.71 (0.40—1.26)
**Gleason Score**				
Low grade	*ref*			
Intermediate grade	5.71* (5.07—6.43)	1.26 (0.82—1.97)	5.70* (5.06—6.42)	1.23 (0.80—1.91)
High grade	5.57* (4.75—6.55)	0.26* (0.17—0.40)	5.57* (4.74—6.53)	0.26* (0.17—0.40)
No Needle Core Biopsy/TURP Performed	0.22* (0.16—0.30)	0.04* (0.23—0.62)	0.22* (0.16—0.31)	0.04* (0.02—0.06)
Unknown	0.43* (0.27—0.68)	0.12* (0.06—0.24)	0.43* (0.27—0.68)	0.12* (0.06—0.24)
**Hospital Characteristics**				
NCI Designated	0.57* (0.48—0.66)	0.91 (0.72—1.16)	0.56* (0.48—0.66)	0.90 (0.71—1.15)
COC Accredited	1.48* (1.24—1.78)	1.80* (1.38—2.35)	1.49* (1.24—1.78)	1.80* (1.38—2.36)
**Hospital Rurality**				
Metropolitan	*ref*			
Non-Metropolitan	0.39* (0.31—0.49)	0.30* (0.21—0.42)	0.39* (0.32—0.49)	0.29* (0.21—0.41)

^*^ = *p* < 0.05

County and date of diagnosis variables were omitted from this table due to page formatting. Hispanic population was compared to non-Hispanic population. Patients with unknown cancer stage were excluded from regression models. Unknown marital status was retained as a separate category

NCI, National Cancer Institute Designated; COC, Commission on Cancer Accreditation

While a greater proportion of NHB men received PCa-related care at a facility with both NCI and CoC accreditation, there were differences in the reception of definitive treatment based on cancer staging. Controlling for covariates, within the early stage subgroup, we found that there were no statistically significant differences in the reception of definitive treatment at an NCI-designated and CoC-accredited facility between NHW and NHB patients (Table 4). Among those within the advanced stage subgroup, however, NHB men were less likely to receive definitive treatment at a facility with both NCI designation and CoC accreditation when compared to NHW patients (adjusted OR 0.34; 95% CI, 0.13 – 0.93). Hispanic patients with advanced staging were similarly less likely to receive definitive care at a facility with both NCI designation and CoC accreditation when compared to non-Hispanic patients (adjusted OR 0.12; 95% CI, 0.01 – 0.78).

**Table 4:** Odds ratio and absolute risk differences of receiving definitive treatment based on cancer risk at cancer facility

		NCI & CoC	CoC Only	Neither NCI nor COC
		OR (95% CI) ARD (95%CI)		
**Early Stage**	NHW	*ref*	*ref*	*ref*
	NHB	1.20 (0.62 – 2.36) 0.03 (-0.60 – 0.12)	0.88 (0.62 – 1.25) -0.02 ( -0.06 – 0.03)	0.53 (0.27 – 1.00) -0.09 (-0.18 – 0.01)
	Hispanic **	0.32 (0.09 – 1.21) -0.17 (0.38 – 0.04)	0.84 (0.39 – 1.82) -0.02 (-0.12 – 0.79)	1.63 (0.39 – 6.91) 0.06 (-0.11 – 0.24)
**Advanced Stage**	NHW	*ref*	*ref*	*ref*
	NHB	0.34* (0.13–0.93) -0.14* (-0.26—-0.01)	0.47*(0.29 – 0.76) -0.10 (-0.17—-0.03)	0.24* (0.10 – 0.60) -0.17* (-0.27—-0.07)
	Hispanic**	0.12* (0.01—0.78) -0.28* (0.52—-0.03)	0.22* (0.10—0.51) -0.21* (-0.33—-0.09)	0.97 (0.22—4.21) -0.01 (-0.18—0.17)

^*^Indicates significance at *p* < 0.05

^**^ non-Hispanic ethnicity was used as the reference category for Hispanic

^†^ Adjusted odds ratio were estimated using logistic regression. Absolute risk differences (ARD) were calculated using post-estimation margins and reflect the adjusted difference in predicted probabilities between comparison groups. Analyses were stratified by cancer risk group and facility accreditation status (NCI designation and or CoC accreditation)

Similarly to facilities with both NCI & CoC status, among patients within the early stage who received definitive care at a facility with only CoC accreditation, after controlling for additional covariates, we found no statistically significant difference in the likelihood of receiving definitive treatment. NHB patients within the advanced stage subgroup were less likely to receive definitive treatment when compared to NHW patients (adjusted OR 0.47; 95% CI, 0.29–0.76). Similarly, Hispanic patients were less likely to receive definitive treatment when compared to non-Hispanic patients (adjusted OR 0.22; 95% CI, 0.10 – 0.51).

At treatment facilities which have attained neither NCI designation nor CoC accreditation, there were no statistically significant differences in the likelihood of receiving definitive care among early stage NHB patients when compared with NHW counterparts after controlling for covariates. Similarly, there were no statistically significant differences among Hispanics within the early stage group compared with non-Hispanics within the early stage. Within the advanced stage group, NHB patients were less likely to receive definitive treatment at a facility which had neither designation nor accreditation (adjusted OR 0.24; 95%CI, 0.10—0.60). There were no statistically significant differences in the likelihood of treatment found between Hispanics and non-Hispanics found within the advanced stage subgroup. For reference, predicted probabilities of definitive treatment by year of diagnosis are presented in the Supplement (Figures S1–S3), corresponding to the odds ratios shown in the main analysis.

#### Time to definitive treatment

We did not find any statistically significant difference in the timeliness of surgical care among either early stage or advanced stage subgroup when comparing NHB patients to NHW patients (Table 5). Similarly, no statistical difference was found among early and advanced stage PCa patients who had radiation as their first course of treatment. Similarly, when comparing Hispanic men to non-Hispanic men, we did not find any differences in timeliness of care for neither early risk nor advanced risk PCa patients. We also conducted additional sensitivity analyses to assess whether disparities in time to definitive treatment emerged when using alternative delay time intervals of 60 and 120 days (Appendix A4 and A5). At the 60-day threshold, unadjusted models showed that NHB men with early stage disease had significantly higher odds of receiving delayed surgery compared to NHW men (OR = 1.93, CI: 1.38–2.70). Additionally, at the 120-day interval, unadjusted models highlighted that NHB men with advanced stage disease were more likely to experience surgery delays greater than 120 days when compared to their NHW counterparts (OR = 1.87, 95% CI: 1.06 – 3.32). However, these time intervals were no longer statistically significant after adjusting for covariates for both time intervals.

**Table 5:** Odds Ratio of NHB vs NHW men receiving surgery or radiation as well as the odds of Hispanic men vs non-Hispanic men receiving surgery or radiation

		NHB vs NHW					Hispanic vs non-Hispanic			
		Unadjusted			Adjusted		Unadjusted		Adjusted	
		Surgery		Radiation	Surgery	Radiation	Surgery	Radiation	Surgery	Radiation
			OR (95% CI)							
Early Stage	< = 90 days	0.82 (0.59–1.15)		0.84 (0.54–1.30)	1.02 (0.73–1.45)	1.10 (0.68–1.78)	0.79 (0.41–1.52)	1.21 (0.46–3.18)	0.78 (0.39–1.57)	1.40 (0.48–4.09)
	> 90 days	1.22 (0.87–1.71)		1.19 (0.77–1.84)	0.98 (0.69 – 1.40)	0.92 (0.57–1.49)	1.22 (0.87–1.71)	0.83 (0.31–2.18)	1.27 (0.64–2.54)	0.70 (0.24–2.03)
Advanced Stage	< = 90 days	0.77 (0.47–1.27)		1.28 (0.31–5.20)	0.92 (0.53–1.59)	0.20 (0.03–1.51)	0.60 (0.11–3.34)	N/A	1.27 (0.52–3.08)	N/A
	> 90 days	1.30 (0.79–2.13)		0.78 (0.19–3.18)	1.02 (0.58–1.77)	4.90 (0.66–36.23)	2.83 (0.51–15.73)	N/A	0.79 (0.32–1.92)	0.70 (0.24–2.03)

^*^Indicates significance at *p* < *0.05*

‘N/A’ an estimate which was not reported due to too few observations (< 10), preventing stable and reliable inference

**Figure 1:**
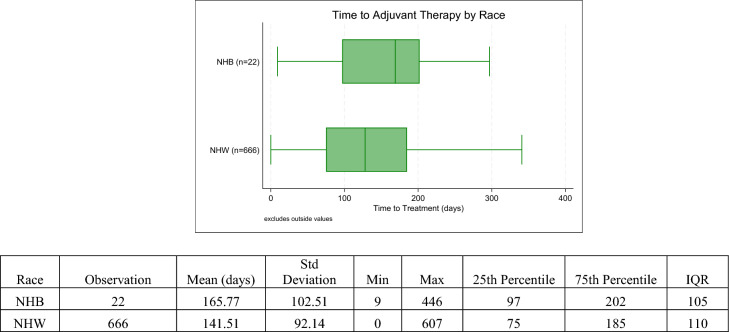
Distribution of Time to Adjuvant Therapy by Race Group. The box plot compares the distribution of time to adjuvant therapy (radiation) in days between NHB (*n* = 22) and NHW (*n* = 666) Iowan men. The mean time to treatment is 165.7 days for NHB with a standard deviation of 102.51 days, and 141.51 days for NHW with a standard deviation of 92.14 days. The minimum and maximum days are 9 and 446 days for NHB, and 0 and 607 days for NHW, respectively. The 25th and 75th percentiles are 97 and 202 days for NHB, and 75 and 185 days for NHW, resulting in interquartile ranges of 105 days for NHB and 110 days for NHW

Among those who received adjuvant therapy, NHB patients were more likely to have a greater time between surgery and the start of their radiation treatment (Fig. 1). On average, NHB patients waited 25 days longer to receive adjuvant radiation treatment when compared to their NHW counterparts. However, this difference was not statistically significant (*p* = 0.16).

## Discussion

In this study, we examined racial and ethnic differences in the receipt and timeliness of prostate cancer (PCa) treatment among NHB, NHW, and Hispanic men in Iowa. We found that NHB men were significantly less likely to receive any form of definitive treatment, regardless of cancer stage. Among those who did receive definitive treatment, there was no statistically significant difference in the time from diagnosis to initiation of surgery for patients whose first course of treatment was surgery. However, differences emerged in the timing of treatment for those who received definitive radiation therapy as their initial treatment modality. When evaluating timeliness more broadly, we found no significant differences between NHB and NHW men in the likelihood of receiving any definitive treatment within the first 90 days of diagnosis.

Our study found racial disparities in receipt of definitive treatment, corroborating with previous studies showing variance in cancer treatment modalities experienced by NHB men when compared to their NHW counterparts [16, 17, 48, 49]. Prior research shows that socioeconomic challenges may contribute to these disparities [10, 50]. However, we found disparities occurred even when accounting for these potential SES differences. Individuals with lower SES may often struggle to receive treatment due to their inability to afford treatment[50]. Within our study, we accounted for the ability to pay based on the possession of health insurance. Our findings further build on previous evidence as we found that even when accounting for potential socioeconomic challenges, there remain differences in the actual treatment that is received by members of various races. Geographic disparities often go in tandem with socioeconomic challenges. While being able to afford the care is essential, being in close proximity to high-quality care is also important in overall survivorship [51]. Distance from the treatment site may play a factor in the course of treatment received and thus the overall survivorship. Previous work suggested that a contributor to poor PCa outcomes for NHB men is due to the longer travel to high-quality care treatment facilities which they experience [52]. This study, however, brings new light to this discourse, as we found that NHB men lived closer to facilities with NCI and CoC statuses than their NHW counterparts and had a greater proportion seeking care at these facilities.

We did not find statistically significant delays in care, but we found that among men who received adjuvant therapy, NHB men were more likely to experience delays in radiation treatment post-surgery. NHB men are more frequently diagnosed with aggressive PCa. While our findings were not statistically significant, the observed 25-day longer median delay among NHB men may still be clinically meaningful. For higher risk profiles, the likelihood of recurrence subsequent to single-modality therapy elevates, underscoring the potential necessity for a combination of treatment modalities to attain optimal outcomes [47]. We hypothesize that both provider-related and patient-related rationale and decision-making may contribute to the observed delays in adjuvant therapy among NHB patients. On the provider side, based on the patient’s case, there might be the recommendation to delay treatment if the adjuvant treatment may not be seen as significantly beneficial to the overall prognosis. As such, the decision may be to actively surveil the site, with close monitoring of the progression through PSA testing. On the other hand, patients may be weary of undergoing another course of treatment, given the possible side effects and quality of life concerns which may be associated with radiation treatment.

We then posit that these differences in treatment and time to treatment be viewed as disparities and not simply differences. A driving point of conversation has been that NHB patients do not receive care at the same health care institutions as NHW patients, and often at lower quality care facilities [53–55]. Our study provides evidence that even when there is similar ability to pay for healthcare services, as well as preferred geographical location, there is a racial disparity in those who receive definitive treatment compared to those who do not. Even after accounting for socioeconomic status, age, residence, and cancer staging, there were differences in the likelihood of receiving treatment. With the statistical difference found only among advanced staging, there may be additional reasons as to why regardless of the quality-of-care status of the facility, NHB men are less likely to receive definitive care compared to NHW patients. Approximately 75% of medical encounters involving NHB patients exhibit racial discordance [56, 57]. Medical interactions between providers and NHB patients may often be unproductive, as these interactions are shorter in length, less patient-centered, and offer less opportunity for a comfortable physician–patient relationship in which trust and understanding may develop [56]. With a lack of trust and comfort within the physician–patient relationship, NHB patients may not completely comprehend the nature of their diagnosis nor treatment options which may be available. Prior research suggests that NHB patients were more likely to not be satisfied with treatment [58]. NHB or African Americans have traditionally reported high levels of medical mistrust. Events such as the Tuskegee syphilis experiment, as well as unethical medical practices on Black boys in which violated human subject policies, have been widely recognized as a reason medical mistrust exists within the NHB community [59, 60]. It is then important that healthcare professionals become culturally aware of NHB patients’ concerns and potential mistrust. Additionally, the American Society for Clinical Oncology recommends additional efforts to reduce structural barriers, such as improving diversity of the oncology workforce, enhancing community partnerships, and strengthening training for providers to improve cancer health equity [61].

Our findings reveal the disparities which exist in both the timing to definitive treatment and likelihood of definitive treatment received between NHB and NHW PCa patients. These disparities highlight the need for critical clinical and health policy improvements in aid of achieving equity in PCa care. Our study highlights the importance of greater implementation and expansion of patient navigation services in healthcare facilities that have a high NHB patient mix. Navigators may assist patients in further understanding their diagnosis, accessing financial assistance, scheduling timely consultations, and improving patient satisfaction [62]. These patient navigator attributes may be key steps in reducing time to treatment disparities and further empowering patients in understanding their course of care. Additionally, with the known benefits of multidisciplinary care teams in oncology practice[63, 64], large cancer centers serving a high proportion of NHB patients should prioritize expanding access to such coordinated care models. The integration of multidisciplinary teams into routine practice may strengthen the shared decision-making process and serve as a structural intervention to reduce treatment delays and improve equity in cancer care. Given the observed racial disparities in treatment modality, medical schools and healthcare organizations should implement ongoing, culturally responsive training for clinicians and staff. Such initiatives are essential for increasing provider awareness of implicit biases and communication behaviors that may be perceived negatively by NHB patients, which will create a more equitable and respectful patient-provider interaction. Finally, it is important that future research further explores the attitudes, perceptions, and barriers leading to reluctance in engaging in oncological care among NHB patients across the cancer care continuum. This potential area of research may offer further insight into the contextual and interpersonal factors which influence treatment decisions and care experiences.

**Figure 2:**
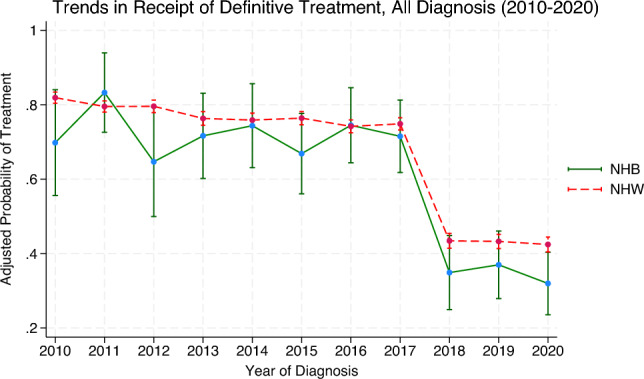
Adjusted predicted probability of receiving definitive prostate cancer therapy by race and year (2010–2020). Figure 2 shows adjusted predicted probability of receiving definitive PCa therapy by race and year of diagnosis (2010–2020) for all diagnoses regardless of stage grouping. Predicted probabilities were estimated using a multivariable logistic regression model adjusting for age group, marital status, Gleason score, cancer staging, insurance status, patient geographical location, hospital rural–urban classification, residence type, NCI designation status, and CoC accreditation. An interaction term between race and year of diagnosis was included to allow for differential trends over time. Results reflect adjusted estimates of the likelihood of receiving definitive treatment among NHB and NHW patients

**Figure 3:**
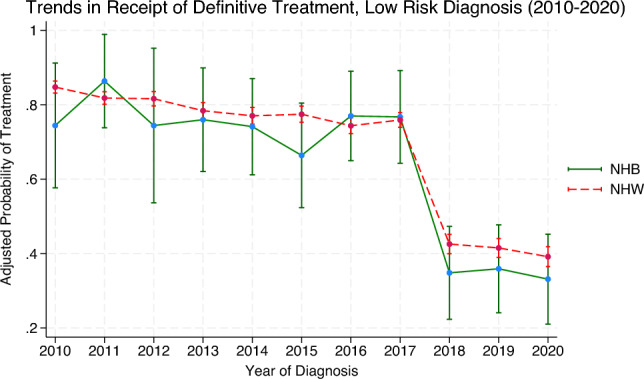
Adjusted predicted probability of receiving definitive prostate cancer therapy by race and year for low-risk cancer diagnosis (2010–2020). Figure 3 depicts the adjusted predicted probability of receiving definitive PCa therapy by race and year of diagnosis (2010=2020) for low-risk diagnosis Predicted probabilities are estimated using a multivariable logistic regression model adjusting for age group, marital status, Gleason score, cancer staging, insurance status, patient geographical location, hospital rural–urban classification, residence type, NCI-designation status, and CoC accreditation. An interaction term between race and year of diagnosis is included to allow for differential trends over time. Results reflect adjusted estimates of the likelihood of receiving definitive treatment among NHB and NHW patients

**Figure 4:**
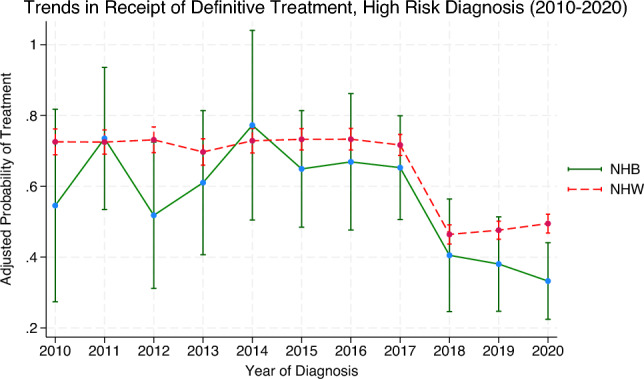
Adjusted predicted probability of receiving definitive prostate cancer therapy by race and year for high-risk cancer diagnosis (2010 -2020). Figure 4 shows adjusted predicted probability of receiving definitive PCa therapy by race and year of diagnosis (2010–2020) for high-risk diagnosis. Predicted probabilities are estimated using multivariable logistic regression model adjusting for age group, marital status, Gleason score, cancer staging, insurance status, patient geographical location, hospital rural–urban classification, residence type, NCI-designation status, and CoC accreditation. An interaction term between race and year of diagnosis is included to allow for differential trends over time. Results reflect adjusted estimates of the likelihood of receiving definitive treatment among NHB and NHW patients

It is also of importance that future research seeks to further understand and find ways in mitigating this reluctancy in receiving medical care from the purview of the NHB patient.(Fig. 2, 3, 4).

### Limitations

This study is not without its limitations. Firstly, our study sample is limited to one state, which may bring into consideration the issue of generalization. However, some of the largest differences in mortality between Iowa’s NHB and NHW populations included deaths related to PCa. Also, our sample size of both NHB as well as Hispanic patients was small. As such, this left our study low on power when seeking statistical inference for subgroups. However, while Iowa’s NHB population is relatively small, these limitations highlight the importance of examining disparities in less diverse, resource variable, and often rural settings where structural barriers to equitable care may be more pronounced but overlooked. This study is also not exempt from potential selection bias, as patients who received definitive treatment may differ systematically from those who did not in ways that are not captured within the data. For example, patient preferences, provider treatment preferences, and attitudes toward PCa, or additional health system-level factors that may influence treatment decisions. Although we adjusted for several clinical and demographic variables, there are potential confounders that were not adjusted for given the data available. For example, information on comorbidities was not captured, which may influence both treatment eligibility and outcomes. Similarly, socioeconomic factors beyond insurance status such as education level, income, and employment may also affect treatment and timing to treatment. The absence of these variables may introduce bias in the estimated associations and hinder our ability to fully explain the underlying factors contributing to observed disparities. Finally, our dataset was only able to capture the first course of treatment for each patient. As such, it is possible that for PCa patients who may have opted for other courses of treatment then proceeded with definitive treatment, their entire course of treatment was not captured.

## Conclusion

This study assesses racial and ethnic disparities in treatment modality and timing of prostate cancer in Iowa. After accounting for demographic, clinical, and treatment characteristics, we found that advanced staged NHB prostate cancer patients were less likely to receive definitive treatment than their NHW counterparts. Our analysis did not find a difference in the timing from diagnosis to initiation of definitive treatment.

Future research should seek to identify the underlying issues related to the delays in treatment and potential barriers. While our research highlighted several differences with treatment and timing of such treatment, we did not assess the outcomes of these treatments. Also, additional research should seek to understand the attitudes and perceptions NHB men have regarding PCa and its impact on their lives. Finally, future researchers should aim to elucidate the level of mistrust in which Black cancer patients may have toward the healthcare systems. This may provide further insight from a patient perspective regarding the reasoning behind the delays in receiving care.

## Supplementary Information

Below is the link to the electronic supplementary material.

Supplementary file1 (DOCX 23 KB)

## Data Availability

Data and materials from this study are not publicly available; however, they may be accessed upon request from the Iowa Cancer Registry.
