# Supplementary material for: Differences in prostate cancer treatment receipt and timeliness of treatment between African American/Black and White Iowans
Source: Cancer Causes Control. 2025 Oct 13;36(12):2001–14. doi: 10.1007/s10552-025-02064-6 (PMC12630164; doi:10.1007/s10552-025-02064-6)
Supplement: Supplementary file 1 — Supplementary file1 (DOCX 23 KB) [file 10552_2025_2064_MOESM1_ESM.docx]

**Appendix A: Supplementary Analysis**

**Figure S1. Adjusted predicted probability of receiving definitive prostate cancer therapy by race and year (2010-2020)**
Figure S1 showing adjusted predicted probability of receiving definitive PCa therapy by race and year of diagnosis (2010-2020) for all diagnosis regardless of stage grouping. Predicted probabilities were estimated using multivariable logistic regression model adjusting for age group, marital status, Gleason score, cancer staging, insurance status, patient geographical location, hospital rural-urban classification, residence type, NCI-designation status, and CoC accreditation. An interaction term between race and year of diagnosis was included to allow for differential trends over time. Results reflect adjusted estimates of the likelihood of receiving definitive treatment among NHB and NHW patients

**Figure S2. Adjusted predicted probability of receiving definitive prostate cancer therapy by race and year for low-risk cancer diagnosis (2010 -2020)**

Figure S2 showing adjusted predicted probability of receiving definitive PCa therapy by race and year of diagnosis (2010-2020) for low-risk diagnosis Predicted probabilities were estimated using multivariable logistic regression model adjusting for age group, marital status, Gleason score, cancer staging, insurance status, patient geographical location, hospital rural-urban classification, residence type, NCI-designation status, and CoC accreditation. An interaction term between race and year of diagnosis was included to allow for differential trends over time. Results reflect adjusted estimates of the likelihood of receiving definitive treatment among NHB and NHW patients

**Figure S3. Adjusted predicted probability of receiving definitive prostate cancer therapy by race and year for high-risk cancer diagnosis (2010 -2020)**

Figure S3 showing adjusted predicted probability of receiving definitive PCa therapy by race and year of diagnosis (2010-2020) for high-risk diagnosis. Predicted probabilities were estimated using multivariable logistic regression model adjusting for age group, marital status, Gleason score, cancer staging, insurance status, patient geographical location, hospital rural-urban classification, residence type, NCI-designation status, and CoC accreditation. An interaction term between race and year of diagnosis was included to allow for differential trends over time. Results reflect adjusted estimates of the likelihood of receiving definitive treatment among NHB and NHW patients

**Table A1: Multivariable analysis of predictors of receiving definitive treatment among NHB men when compared to NHW including interaction of NHB and hospitals based on characteristics.**

|  | Early Stage (n=12,342) | Advanced Stage (n=5,187) | Early Stage (n=12,342) | Advanced Stage (n=5,187) |
| --- | --- | --- | --- | --- |
|  | NHB compared to NHW | | Hispanic compared to Non-Hispanic | |
|  | OR (95%CI) | | | |
| **Race/Ethnicity** |  |  |  |  |
| NHW | *ref* |  |  |  |
| NHB | 0.43* (0.24 – 0.78) | 0.23* (0.10 – 0.55) |  |  |
| Non-Hispanic |  |  | *ref* |  |
| Hispanic |  |  | 3.09 (0.56 – 17.10) | 0.39* (0.18 – 0.84) |
| **Hospital Characteristics** |  |  |  |  |
| NCI Designated | 0.56* (0.48 - 0.66) | 1.17 (0.91 – 1.52) | 0.13* (0.03 - 0.62) | 1.18 (0.92 – 1.53) |
| COC Accredited | 1.38* (1.14 – 1.67) | 1.50* (1.12 – 2.01) | 1.43* (1.19 - 1.73) | 1.50* (1.12 – 2.01) |
| NHB x NCI Designated | 1.04 (0.49 – 2.19) | 0.99 (0.33 – 2.94) |  |  |
| NHB x COC Accredited | 2.05 (0.89 – 4.23) | 2.25 (0.79 – 6.40) |  |  |
| Hispanic x NCI Designated |  |  | 0.13* (0.03 – 0.62) |  |
| Hispanic x COC Accredited |  |  | 0.54* (0.08 – 3.83) |  |
| **Age Groups** |  |  |  |  |
| <50 | *ref* |  |  |  |
| 50-64 | 0.75 (0.47 – 1.20) | 0.80 (0.36 – 1.75) | 0.75 (0.47 – 1.20) | 0.78 (0.36 – 1.72) |
| 65-74 | 0.43* (0.27 - 0.70) | 0.46 (0.20 – 1.03) | 0.43* (0.27 - 0.71) | 0.44 (0.20 – 1.00) |
| 75+ | 0.09* (0.06 – 0.15) | 0.09* (0.04 – 0.21) | 0.09* (0.06 - 0.16) | 0.09* (0.04 - 0.20) |
| **Marital Status** |  |  |  |  |
| Married | *Ref* |  |  |  |
| Separated/Divorced/Widowed | 0.63* (0.54 - 0.74) | 0.74* (0.59 – 0.94) | 0.63* (0.54 - 0.74) | 0.74* (0.59 - 0.94) |
| Single, Never Married | 0.60* (0.50 - 0.72) | 0.54* (0.41 - 0.72) | 0.60* (0.50 - 0.71) | 0.55* (0.41 - 0.72) |
| Unknown | 0.73* (0.06 - 0.15) | 0.90 (0.52 – 1.53) | 0.73* (0.54 – 0.98) | 0.89 (0.52 – 1.52) |
| **Insurance Status** |  |  |  |  |
| Government Military | *Ref* |  |  |  |
| Insurance, NOS | 2.55* (1.82 – 3.58) | 2.14* (1.15 – 3.97) | 2.60* (1.85 - 3.65) | 2.14* (1.15 – 3.97) |
| Medicaid | 1.89* (1.20 - 2.97) | 0.94 (0.50 – 1.75) | 1.87* (1.19 - 2.93) | 0.92 (0.49 – 1.73) |
| Medicare | 1.61* (1.25 - 2.07) | 1.25 (0.82 - 1.90) | 1.63* (1.27 - 2.10) | 1.25 (0.82 - 1.90) |
| Private | 2.25* (1.72 – 2.93) | 1.49 (0.94 - 2.37) | 2.29* (1.75 – 2.99) | 1.49 (0.94 – 2.36) |
| None/Unknown | 1.93* (1.32 – 2.83) | 0.65 (0.35 - 1.20) | 1.95* (1.33 - 2.85) | 0.67 (0.36 - 1.25) |
| **Gleason Score** |  |  |  |  |
| Low grade | *ref* |  |  |  |
| Intermediate grade | 1.56* (1.32 – 1.84) | 1.76* (1.11 – 2.81) | 1.56* (1.32 – 1.84) | 1.78* (1.12 – 2.83) |
| High grade | 1.49* (1.22 – 1.81) | 0.55* (0.35 - 0.87) | 1.49* (1.22 - 1.81) | 0.55* (0.35 - 0.86) |
| No Needle Core Biopsy/TURP Performed | 0.13* (0.09 - 0.18) | 0.16* (0.10 - 0.28) | 0.13* (0.09 – 0.18) | 0.16* (0.10 - 0.27) |
| Unknown | 0.32* (0.20 - 0.53) | 0.36* (0.17 - 0.77) | 0.32* (0.20 - 0.53) | 0.36* (0.17 - 0.77) |
| **Hospital Rurality** |  |  |  |  |
| Metro | *ref* |  |  |  |
| Non-Metro | 0.38* (0.31 – 0.48) | 0.32* (0.23 - 0.46) | 0.39* (0.31 - 0.49) | 0.32* (0.22 - 0.46) |
| x = Denotes interaction between independent variables * = p <0.05 Note: County and date of diagnosis variables were omitted from this table due to page formatting . Hispanic population was compared to non-Hispanic population. Patients with unknown cancer stage were excluded from regression models. Unknown marital status was retained as a separate category | | | | |

**Table A2: Absolute Risk Difference (ARD) of NHB vs NHW men receiving surgery or radiation as well as the odds of Hispanic men vs non-Hispanic men receiving surgery or radiation**

45jklhjk

|  |  | NHB vs NHW | |  | | Hispanic vs non-Hispanic | | | |
| --- | --- | --- | --- | --- | --- | --- | --- | --- | --- |
|  |  | Unadjusted | | Adjusted | | Unadjusted | | Adjusted | |
|  |  | **Surgery** | **Radiation** | **Surgery** | **Radiation** | **Surgery** | **Radiation** | **Surgery** | **Radiation** |
| OR (95% CI) | | | | | | | | | |
| Early Stage | <= 90 days | 0.01 (0.59-1.15) | -0.03 (-0.13 – 0.08) | 0.01 (-0.06 – 0.07) | 0.03 (-0.07 – 0.14) | -0.03 (-0.18 – 0.12) | 0.03 (-0.20 – 0.26) | -0.06 (-0.19 – 0.08) | 0.07 (-0.15 – 0.29) |
|  | >90 days | 0.05 (-0.02 – 0.12) | 0.03 (-0.08 – 0.13) | -0.01 (-0.07 - 0.06) | -0.03 (-0.14 – 0.07) | 0.05 (-0.09 – 0.19) | -0.03 (-0.26 – 0.20) | 0.03 (-0.10 – 0.16) | -0.07 (-0.29 – 0.15) |
| Advanced Stage | <= 90 days | -0.04 (-0.15 – 0.06) | -0.05 (-0.43 – 0.33) | -0.01 (-0.11 – 0.08) | -0.12 (-0.30 – 0.06) | 0.06 (-0.08 – 0.20) | -0.03 (-0.26 – 0.20) | 0.05 (-0.09 – 0.19) | -0.14 (-0.52 – 0.24) |
|  | >90 days | 0.05 (-0.05 – 0.15) | 0.05 (-0.32 – 0.43) | -0.01 (-0.09 - 1.77) | 0.13 (-0.05 – 0.30) | -0.04 (-0.17 – 0.10) | -0.05 (0.43 – 0.33) | -0.04 (-0.16 – 0.09) | 0.14 (-0.24 – 0.51) |

* = p <0.05  **Table A2.** Absolute Risk Difference comparing the likelihood of receiving surgery or radiation within or beyond 90 days of diagnosis among NHB vs NHW men, and Hispanic vs non-Hispanic men, stratified by cancer stage and treatment type. Adjusted models controlled for sociodemographic and clinical covariates. Negative ARD values indicate a lower predicted probability of receiving treatment within the time frame when compared to the reference group (NHW or Hispanic)

45jklhjk**Table A3 Odds Ratio of NHB vs NHW men receiving surgery or radiation as well as the odds of Hispanic men vs non-Hispanic men receiving surgery or radiation after 60 days post diagnosis**

| Treatment | Stage | Groups | OR (95%CI) Unadjusted | OR(95%CI) Adjusted | ARD (95%CI) Adjusted |
| --- | --- | --- | --- | --- | --- |
| Surgery | Early | NHB vs NHW | 1.93* (1.38 -2.70) | 1.39 (0.96 – 2.00) | 0.07 (-0.01 – 0.14) |
| Surgery | Early | Hispanic vs non-Hispanic | 1.75 (0.92 – 3.30) | 1.70 (0.85 – 3.37) | 0.10 (-0.02 – 2.31) |
| Surgery | Advanced | NHB vs NHW | 1.44 (0.91 – 2.26) | 1.08 (0.65 – 1.80) | 0.02 (-0.09 – 0.13) |
| Surgery | Advanced | Hispanic vs non-Hispanic | 1.33 (0.67 – 2.62) | 1.39 (0.67 – 2.87) | 0.07 (-0.08 – 0.22) |
| Radiation | Early | NHB vs NHW | 1.10 (0.67 – 1.81) | 0.93 (0.53 – 1.61) | -0.01 (-0.11 – 0.08) |
| Radiation | Early | Hispanic vs non-Hispanic | 1.77 (0.51 – 6.15) | 1.80 (0.47 – 6.90) | 0.09 (-0.09 – 0.26) |
| Radiation | Advanced | NHB vs NHW | 0.95 (0.34 – 2.68) | 1.56 (0.31- 7.71) | 0.05 (-0.11 – 0.20) |
| Radiation | Advanced | Hispanic vs non-Hispanic | 1.71 (0.20 – 14.75) | 1.21 (0.08 – 18.14) | 0.20 (-0.26 – 0.30) |

* = p <0.05
**Table A3,** Odds Ratios (Ors) and Absolute Risk Differences (ARDs) for receiving surgery or radiation more than 60 days after diagnosis, comparing NHB vs NHW men and Hispanic vs non-Hispanic men. Results are stratified by cancer stage (early vs advanced) and treatment modality (surgery vs radiation). Unadjusted and adjusted ORs are presented as well as adjusted ARDs derived from predictive margins. Positive ARDs indicate a higher predicted probability of treatment delay in the comparison group relative to the reference. Adjusted models controlled for sociodemographic, clinical and facility level characteristics specified in the methods section.

45jklhjk

**Table A4 Odds Ratio of NHB vs NHW men receiving surgery or radiation as well as the odds of Hispanic men vs non-Hispanic men receiving surgery or radiation after 120 days post diagnosis**

| Treatment | Stage | Groups | OR (95%CI) Unadjusted | OR(95%CI) Adjusted | ARD (95%CI) Adjusted |
| --- | --- | --- | --- | --- | --- |
| Surgery | Early | NHB vs NHW | 1.18 (0.77 – 1.79) | 0.90 (0.58 – 1.40) | -0.01 (-0.06 – 0.04) |
| Surgery | Early | Hispanic vs non-Hispanic | 1.35 (0.63 – 2.92) | 1.70 (0.85 – 3.37) | 0.10 (-0.023 – 0.23) |
| Surgery | Advanced | NHB vs NHW | 1.87* (1.06 – 3.32) | 1.67 (0.88 – 3.15) | 0.06 (-0.02 – 0.14) |
| Surgery | Advanced | Hispanic vs non-Hispanic | 0.23 (0.30 – 1.72) | 1.39 (0.67 – 2.87) | 0.07 (-0.08 – 0.22) |
| Radiation | Early | NHB vs NHW | 1.35 (0.86 – 2.13) | 1.05 (0.64 – 1.71) | 0.01 (-0.08 – 0.10) |
| Radiation | Early | Hispanic vs non-Hispanic | 1.01 (0.36 – 2.85) | 0.86 (0.28 – 2.66) | -0.03 (-0.22 – 0.17) |
| Radiation | Advanced | NHB vs NHW | 1.82 (0.71 – 4.68) | 2.70 (0.70 – 10.43) | 0.15 (-0.04 – 0.35) |
| Radiation | Advanced | Hispanic vs non-Hispanic | 1.06 (0.21 – 5.22) | 1.33 (0.11 - 16.06) | 0.05 (-0.35 – 0.44) |

* = p <0.05  **Table A4,** Odds Ratios (Ors) and Absolute Risk Differences (ARDs) for receiving surgery or radiation more than 120 days after diagnosis, comparing NHB vs NHW men and Hispanic vs non-Hispanic men. Results are stratified by cancer stage (early vs advanced) and treatment modality (surgery vs radiation). Unadjusted and adjusted ORs are presented as well as adjusted ARDs derived from predictive margins. Positive ARDs indicate a higher predicted probability of treatment delay in the comparison group relative to the reference. Adjusted models controlled for sociodemographic, clinical and facility level characteristics specified in the methods section.
